# The paleoenvironments of azhdarchid pterosaurs localities in the Late Cretaceous of Kazakhstan

**DOI:** 10.3897/zookeys.483.9058

**Published:** 2015-02-20

**Authors:** Alexander Averianov, Gareth Dyke, Igor Danilov, Pavel Skutschas

**Affiliations:** 1 Zoological Institute of the Russian Academy of Sciences, Universitetskaya nab. 1, 199034 Saint Petersburg, Russia; 2 Department of Sedimentary Geology, Geological Faculty, Saint Petersburg State University, 16 liniya VO 29, 199178 Saint Petersburg, Russia; 3 Ocean and Earth Science, National Oceanography Centre, Southampton, University of Southampton, Southampton SO14 3ZH, UK; 4 MTA-DE Lendület Behavioural Ecology Research Group, Department of Evolutionary Zoology and Human Biology, University of Debrecen, 4032 Debrecen, Egyetem tér 1, Hungary; 5 Zoological Institute of the Russian Academy of Sciences, Universitetskaya nab. 1, 199034 Saint Petersburg, Russia; 6 Department of Vertebrate Zoology, Biological Faculty, Saint Petersburg State University, Universitetskaya nab. 7/9, 199034 Saint Petersburg, Russia

**Keywords:** Pterosauria, Azhdarchidae, Late Cretaceous, Kazakhstan, distribution, paleoenvironments

## Abstract

Five pterosaur localities are currently known from the Late Cretaceous in the northeastern Aral Sea region of Kazakhstan. Of these, one is Turonian-Coniacian in age, the Zhirkindek Formation (Tyulkili), and four are Santonian in age, all from the early Campanian Bostobe Formation (Baibishe, Akkurgan, Buroinak, and Shakh Shakh). All so far collected and identifiable Late Cretaceous pterosaur bones from Kazakhstan likely belong to Azhdarchidae: *Azhdarcho* sp. (Tyulkili); *Aralazhdarcho bostobensis* (Shakh Shakh); and *Samrukia nessovi* (Akkurgan). These latter two taxa, both from the Bostobe Formation might be synonyms. *Azhdarcho* sp. from the Zhirkindek Formation lived in a tropical-to-subtropical relatively humid climate on the shore of an estuarine basin connected to the Turgai Sea. Known fossils were collected in association with brackish-water bivalves and so the overall paleoenvironment of this pterosaur was likely an estuarine marsh as indicated by the dominance of conifers and low relative counts of ferns and angiosperms. *Aralazhdarcho bostobensis*, from the Bostobe Formation, lived on a coastal fluvial plain along the Turgai Sea. This paleoenvironment was either floodplain (Akkurgan, Buroinak, and Shakh Shakh) or estuarine (Baibishe). In the Santonian – early Campanian, shallow waters near this coastal plain were sites for the intensive accumulation of phosphates under upwelling conditions caused by strong winds from the ancient Asian landmass. These winds also caused significant aridization of the climate during this time. We speculate that pterosaurs may have been attracted to this area by the abundant resources in the bio-productive estuaries and nearshore upwelling waters.

## Introduction

**Figure 1.**
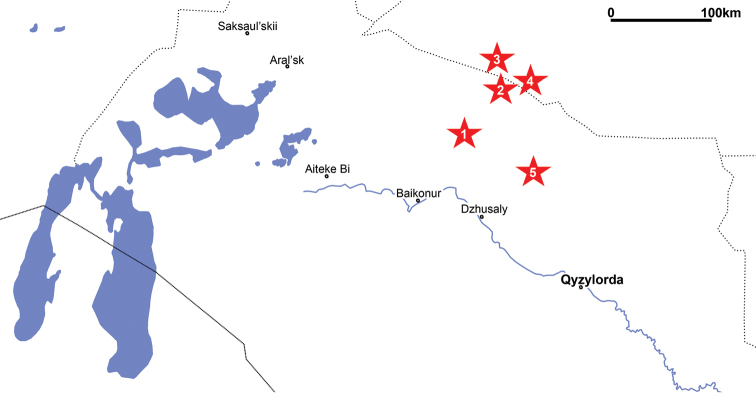
Map to show the northeastern Aral Sea region of Kazakhstan and the approximate positions of known Late Cretaceous pterosaur localities (**1** Tyulkili **2** Baibishe **3** Akkurgan **4** Buroinak **5** Shakh-Shakh). The lakes in the western part of the map are remnants of the Aral Sea, relics of the Turgai Strait that once connected the Tethys and Arctic oceans.

In Kazakhstan two regions are known to have yielded the skeletal remains of pterosaurs: 1) the Upper Jurassic (Oxfordian-Kimmeridgian) Karabastau Formation in the Karatau Mountains of southern Kazakhstan, and; 2) several sites within the Late Cretaceous Zhirkindek (Turonian-Coniacian) and Bostobe (Santonian – lower Campanian) formations in the northeastern Aral Sea region of western Kazakhstan (Fig. 1). Of these, the latter has yielded isolated bones of large pterodactyloid pterosaurs while the former is a Konservat-Lagerstätte known to contain exceptionally well-preserved skeletons with soft tissues of the non-pterodactyloid pterosaurs *Batrachognathus volans* and *Sordes pilosus* (Riabinin 1948; Sharov 1971; Unwin and Bakhurina 1994).

The first pterosaur bones from the northeastern Aral Sea region of Kazakhstan were described by Nesov (1984). Subsequently, two taxa have been described from the Bostobe Formation: *Aralazhdarcho bostobensis* (Averianov 2004, 2007a) and *Samrukia nessovi* (Buffetaut 2011; Naish et al. 2012). Here we review all previous finds of pterosaurs from the northeastern Aral Sea region, report on additional specimens collected on our expedition in 2012, and discuss the paleoenvironment of the pterosaurs that lived in this region.

Institutional abbreviations: CCMGE, Chernyshev’s Central Museum of Geological Exploration, Saint Petersburg, Russia; SMNK PAL, Staatliches Museum für Naturkunde, Karlsruhe, Germany; WDC, the Wyoming Dinosaur Center, Thermopolis, USA; ZIN PH and ZIN PO, Paleoherpetological and Paleoornithological collections of the Zoological Institute of the Russian Academy of Sciences, Saint Petersburg, Russia.

## Late Cretaceous pterosaur localities in Kazakhstan

### Tyulkili

The Tyulkili [=Tjulkeli] hills (known in the paleobotanical literature as the Kankazgan locality) are located about 80 km north of Dzhusaly railway station in northeastern Kazakhstan (Fig. 1). Here the Zhirkindek Formation is exposed and is composed of sands interbedded with clays and silts (Shilin 1998; Kordikova et al. 2001). The ferruginous sandstone at the bottom of the Zhirkindek Formation section at Tyulkili hills has yielded numerous plant remains, including 36 species of fossil angiosperms (Shilin 1982, 1986, 1998). The next bed up-sequence, consisting of yellow-grey and grey clays, represents an ingression of brackish waters. It has produced fossilized wood, numerous remains of crustaceans, shark teeth (*Protolamna* sp., *Scapanorhynchus* sp.) and coprolites, the isolated bones of lindholmemydid and trionychid turtles, crocodyliforms, ornithopods (Hadrosauroidea indet.), possible sauropods, and laterally compressed teeth of theropods (Tyrannosauroidea indet.) (Nesov 1995, 1997). Indeed, in 2012 the tooth of a marine shark *Cretodus longiplicatus* was found at this level, a species characteristic of the Cenomanian of the Tethys region (Werner 1989; Kennedy et al. 2008; Underwood et al. 2011). According to Sokolov (1978), this species [=*Semiplicatodus sulcatus* in his usage] is widely distributed in late Albian – Cenomanian deposits of the Mangyshlak Peninsula and western Turkmenistan but is rare in the Turonian of Kazakhstan.

The main fossiliferous horizon at the Tyulkili locality is confined to the middle sandstone bed, about 18 m above the base of the Zhirkindek Formation (Kordikova et al. 2001). In 1982, Nesov collected the remains of plesiosaurs, crocodyliforms, pterosaurs (cf. Azhdarchidae) and dinosaurs (Ankylosauridae indet., Hadrosauroidea indet., Neoceratopsia indet., Sauropoda indet., Tyrannosauroidea indet., Ornithomimidae indet., and Therizinosauroidea indet.) at this level (Nesov 1995, 1997; Averianov 2007b, 2009; Averianov and Sues 2009). Martinson (1990b, 1997) reported six species of bivalves (Trigonioidea and Unionoidea) from unspecified levels of the Zhirkindek Formation at Tyulkili (Kankazgan). Much later, Kordikova et al. (2001) reported gastropods, bivalves, brachiopods, crustaceans, selachians, osteichthyans, amphibians, varanoid lizards, trionychid turtles, crocodyliforms (cited as “*Turanosuchus aralensis*” [=*Kansajsuchus borealis*]) and ornithomimid, tyrannosaurid, and dromaeosaurid theropods from this horizon. In 2012, we collected a microvertebrate sample from this level that included shark teeth and spines (*Hybodus* sp., *Polyacrodus* sp.), numerous gar scales and bones (Lepisosteidae indet.), rare dinosaur teeth and salamander bones (Skutschas 2013). Further up in the section, at the top part of the middle sandy bed, there are also shark teeth and spines (Hybodontidae indet., Odontaspididae indet., *Scapanorhynchus* sp.), holostean (Lepisosteidae indet.), teleostean bones and scales, bones of lindholmemydid and trionychid turtles, crocodyliforms, dinosaurs, and fragments of pterosaur hollow bones (Nesov 1997). The upper part of the Zhirkindek Formation at Tyulkili hills is composed of light grey clays from which abundant plant remains as well a bird feather have been collected (Shilin 1986: fig. 3; Nesov 1992a).

The flora known from the lowermost sandstone bed of the Zhirkindek Formation at Tyulkili is dominated by *Platanus* species and thermophilous conifers (Shilin 1998) and likely corresponds to the early Turonian thermal maximum (Nesov 1993). A similar Turonian flora has been collected from the Zhirkindek Formation at Karakumzholy at the eastern end of the Tyulkili hills (Shilin 2008). East from the Tyulkili hills, in the lower Syr Darya area below the Zhirkindek Formation, there are marine early Turonian deposits with inoceramids and fossilized wood (Geology of the USSR 1970). The lower clayey part of the Zhirkindek Formation corresponds to the early Turonian marine transgression in the region and the upper sandy part may be of the late Turonian – Coniacian age (Nesov 1997; Kordikova et al. 2001).

**Figure 2.**
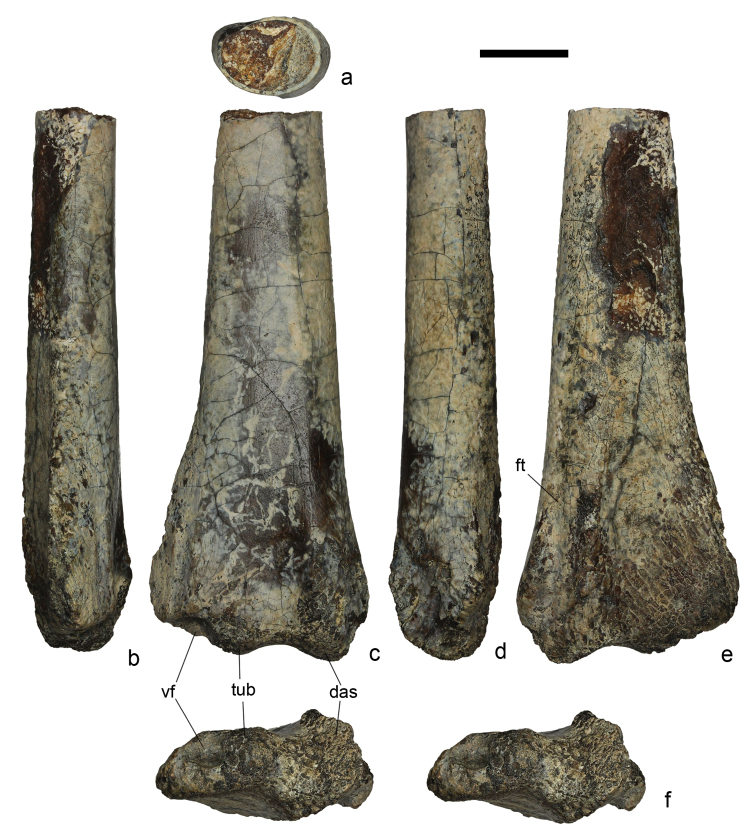
*Azhdarcho* sp., ZIN PH 56/43, distal fragment of a right ulna in proximal (**a**), ventral (**b**), posterior (**c**), dorsal (**d**), anterior (**e**), and distal (**f**, stereopair) views. This specimen is from the Tyulkili locality in the northeastern Aral Sea region of Kazakhstan; Zhirkindek Formation, Upper Cretaceous (upper Turonian – Coniacian). Abbreviations: das, dorsal articulation surface; ft, groove for flexor tendon; tub, tuberculum; vf, ventral fovea. Scale bar is 10 mm.

The pterosaur specimens collected by Nesov in 1982 at Tyulkili include ZIN PH 54/43, a dorsal vertebra (Averianov 2007a: fig. 1), ZIN PH 38/43, a poorly preserved distal fragment of radius or ulna and ZIN PH 13/43, a small fragment of the first wing phalanx (?). In 2012 a well preserved distal fragment of juvenile ulna (ZIN PH 56/43; Fig. 2) was also collected from the lower part of this section. This specimen is about half the size of ZIN PH 14/44, a distal ulna fragment of *Azhdarcho lancicollis* from the upper Turonian Bissekty Formation of the Kyzylkum Desert, Uzbekistan (Averianov 2010: fig. 25F–J): its distal width is 25.3 mm compared with 41.0 mm in the specimen from the Bissekty Formation. ZIN PH 14/44 could be from an adult but is not the largest specimen from the Bissekty Formation as ZIN PH 86/44, an ulna lacking its distal end, is distinctly larger. In spite of these size differences, the morphology of both bones is almost identical: in each the shaft is hollow, with a maximum wall thickness around 1 mm, and is oval in cross section with a dorsoventral long axis (Fig. 2a). The shaft flares towards the distal end and is more pronounced on the ventral side compared with the dorsal side; the distal end is more than twice as expanded dorsoventrally compared with the preserved proximal end. The distal articulation surface for the proximal syncarpal is skewed towards the longitudinal axis of the shaft at an angle ~76°. This surface is composed of the dorsal articulation surface, middle tuberculum, and ventral fovea (Fig. 2). The dorsal articulation surface is slightly convex and crescentic in distal view (Fig. 2f) and has a tongue-like extension on its posterior surface. This extension occupies almost half of the distal articulation surface of the bone. The tuberculum is relatively small and similar in the area to the ventral fovea. In ZIN PH 14/44 (Averianov 2010: fig. 25G, J), this tuberculum appears larger but the ventral fovea is incompletely preserved. On the posterior side of the distal end, ventral to the ventral fovea, there is a short but prominent longitudinal groove and a deeper and longer groove is also positioned on the dorsal margin of the distal end on the anterior side (Fig. 2e), possibly for a flexor tendon (Bennett 2001). Ventrally this groove is bordered by a short prominent ridge, ventral from which the anterior surface is depressed. A similar ridge is present on the opposite posterior side that connects distally with a tongue-like extension of the dorsal articulation surface. Some parts of this ridge and dorsal articulation surface are missing in ZIN PH 56/43.

The morphology of the distal part of the ulna generally shows little variation among pterodactyloid pterosaurs (see review in Wiffen and Molnar 1988). In Ornithocheiridae, the distal articulation surface of the ulna is not skewed and the ventral portion of the distal ulna is usually wider anteroposteriorly with the ventral fovea occupying only half of this width. A pneumatic foramen can often be present on the anterior side of this element close to the distal end (Wellnhofer 1985: fig. 9a, c). In ZIN PH 56/43, common to azhdarchids, this pneumatic foramen is absent and the ventral part of the distal ulna is very narrow, with a ventral fovea occupying most of its surface (Averianov 2010). In non-azhdarchid azhdarchoids, such as “*Santanadactylus spixi*” [=*Tupuxuara* sp.] (Wellnhofer 1985: fig. 25a-c) and *Tapejara wellnhoferi* (SMNK PAL 1137, pers. obs. by AA) the ventral part of the distal ulna is similarly narrow, but the distal articulation surface is less skewed. Because ZIN PH 56/43 is almost undistinguishable from the distal ulna of *Azhdarcho lancicollis* from the co-eval Bissekty Formation in the nearby Kyzylkum Desert, Uzbekistan, it is tentatively assigned here to *Azhdarcho* sp.

### Baibishe

The Baibishe [=Baybishe] hill locality is situated about 130 km NNW from Dzhusaly railway station (Fig. 1). The vertebrate remains from this region come from the lower and middle part of the Bostobe Formation which is exposed here above the upper Albian – Cenomanian Altykuduk Formation and Turonian Zhirkindek Formation, both of which contain plants and molluscs (Martinson et al. 1966; Shilin and Romanova 1978). The first vertebrates from the Bostobe Formation at this locality were discovered by Shilin in 1979 who collected plant remains and molluscs from the lower clay bed, a nearly complete carapace of the adocid turtle *Shachemys baibolatica* and hadrosauroid bones from the overlying yellow-grey sandstone bed (Kuznetsov and Shilin 1983; Shilin 1983). In 1982, Nesov collected a microvertebrate sample from this locality which included brackish water chondrychthians (Polyacrodus cf. brabanticus, *Polyacrodus* sp., *Hybodus kansaiensis*, *Scapanorhynchus* sp., *Ischyrhiza* sp., *Protoplatyrhina* sp., *Ptychotrigon* sp., *Myledaphus glickmani*, *Baibishia baibishe*) and osteichthyans (Ichthyodectidae indet.), freshwater chondrosteans (Acipenseridae indet.) and holosteans (Amiidae indet.), salamanders (*Eoscapherpeton* sp.) and frogs, macrobaenids *Anatolemys maximus*, freshwater adocids *Adocus bostobensis* and *Shachemys baibolatica* and Trionychidae indet., remains of the terrestrial stem testudinoid *Lindholmemys* sp., crocodyliforms cf. *Kansajsuchus* sp., ornithopods, sauropods, small non-avian theropods, tyrannosauroids, and birds (Mertinene and Nesov 1985; Nesov and Mertinene 1986; Nesov 1988a, 1995, 1997; Syromyatnikova and Danilov 2009; Danilov et al. 2011; Skutschas 2013). *Bishara backa*, for example, was based on an atlas vertebra from Baibishe and was first described as an albanerpetontid (Nesov 1997) and then later considered to be an unidentifiable salamander by Gardner and Averianov (1998). This element could be the oldest record of the Proteidae (Skutschas 2013). Nesov (1995) also recorded remains of a possible ceratopsid dinosaur at this site but this cannot be confirmed. A fragmentary skull roof from Baibishe referred by Nesov (1988a) to a hadrosauroid cf. *Lophorothon* turns out to be a giant freshwater trionychid turtle, *Khunnuchelys* n. sp. (Vitek and Danilov 2010; Danilov et al. in press). Averianov (2007b) identified bones and teeth of Tyrannosauroidea indet., Therizinosauroidea indet., and Dromaeosauridae indet. from Baibishe and referred CCMGE 601/12457, a femur from this site attributed by Nesov (1995) to *Tarbosaurus*, to a therizinosauroid cf. *Neimongosaurus* sp. This latter identification has been questioned by Zanno (2010).

A rich assemblage of brackish water bivalves from Baibishe was described by Martinson (1990b): *Lancedaria angustata*, *Psorula tasaranica*, *Parreysia convexa*, *Oxynaia baibishensis*, and *Rectidens asiaticus*. Nesov (1988c) also reported silicified wood at this site that contains the boring traces of insect larvae, likely jewel beetles (Buprestidae) and other coleopterans, which he considered to be ichnospecies *Rhombichnithes beibishensis*.

In the microvertebrate sample from Baibishe there are also a number of indeterminant pterosaur hollow limb bone fragments (Nesov 1990; Averianov 2008). A single large specimen cf. ?Azhdarchidae was reported from this site by Nesov (1997: 111). ZIN PO 3475, a pedal ungual phalanx, is so far the only bird fossil reported from Baibishe (Nesov 1988b: 121, fig. 1(5); 1992b: 27; 1992a: 471, fig. 4K) although Bakhurina and Unwin (1995: 231) noted similarity with bones of *Dsungaripterus* and thought it instead may belong to a pterosaur.

### Akkurgan

Akkurgan [=Akkurgan-Boltyk] is an isolated hill 135 km north of Dzhusaly railway station (Fig. 1) that exposes outcrops of the Bostobe Formation. This locality has become known for the plant remains collected from the light-grey clays at the bottom of the Bostobe Formation (Martinson et al. 1966; Shilin and Romanova 1978). In 1977, Shilin collected plant remains from this level and a hadrosauroid maxilla and femur fragments from the green-grey clays above. This maxilla fragment became the type specimen of *Arstanosaurus akkurganensis*, which is now considered a *nomen dubium* (Shilin and Suslov 1982; Norman and Kurzanov 1997; Horner et al. 2004). In 1982, Nesov collected bones of the freshwater chondrostean *Acipenser shilini*, the adocids *Adocus bostobensis* and *Shachemys baibolatica*, an indeterminate trionychid, the stem testudinoid *Lindholmemys* sp., and crocodyliforms, Hadrosauroidea indet. remains and the laterally compressed teeth of Tyrannosauroidea indet. (Nesov and Kaznyshkin 1983; Nesov 1995, 1997; Syromyatnikova and Danilov 2009; Danilov et al. 2011). Much later, Godefroit et al. (2012) described the basal hadrosauroid *Batyrosaurus rozhdestvenskyi* based on a partial skeleton from this locality.

Most recently, Naish et al. (2012) described a posterior mandible fragment from Akkurgan which they referred to the gigantic bird *Samrukia nessovi*. This specimen was subsequently considered to be a pterosaur (Buffetaut 2011), a reassignment agreed with in this paper. The *Samrukia* mandible is similar to the mandible of *Quetzalcoatlus* sp. from the Maastrichtian of Texas, USA (Kellner and Langston 1996: fig. 4) in having a peculiar posterolateral process of the lateral cotyle of the mandibular glenoid, a feature absent in ornithocheirids and pteranodontids (Wellnhofer 1985; Bennett 2001). Our view is that most likely *Samrukia nessovi* is an azhdarchid pterosaur and thus this name is a possible synonym of *Aralazhdarcho bostobensis* known from the same formation at Shakh Shakh.

### Buroinak

Buroinak [=Boroinak] is a low ridge 110–120 km NNE of Dzhusaly railway station (Fig. 1) that exposes outcrops of the Bostobe Formation. In 1982, Nesov collected the laterally compressed teeth of Tyrannosauroidea indet. from this locality along with other remains of non-avian theropods and the bones of ankylosaurs, possible ceratopsians and hadrosauroids (Nesov 1995). Averianov (2007b) identified Tyrannosauroidea indet. and Therizinosauroidea indet. from within this sample. The other known vertebrates from this site are amiid fishes, macrobaenid, lindholmemydid, adocid, and trionychid turtles, and crocodyliforms (Nesov 1997). The adocid turtle from this locality has been recently identified as *Adocus bostobensis* (Syromyatnikova and Danilov 2009). Martinson (1990b) reported the presence of the brackish water bivalve *Sainshandia syrdarjensis* from this site and silicified wood with beetle larvae borings, *Rhombichnithes boroinakensis*, is also known to occur (Nesov 1988c).

A fragment of a possible pterosaur first wing phalanx has also been collected from the middle part of the Bostobe Formation in the northern part of the Buroinak Ridge (Glickman et al. 1987; Nesov 1990; Bakhurina and Unwin 1995; Nesov 1997; Unwin et al. 1997).

### Shakh-Shakh

Shakh Shakh [=Baibolat, =Zhalmouz] was the first known and now the best sampled vertebrate locality from within the Bostobe Formation. There are two main collecting areas, Shakh Shakh I and II (Rozhdestvensky 1964), separated by about 8 km and located about 70 km north of Dzhusaly railway station (Fig. 1). The locality was discovered by geologists from the Moscow Geological Institute in 1956 (Nikiforova 1960; Rozhdestvensky 1964). In 1957 and 1961 intensive fieldwork was carried out at this locality by teams from the Paleontological Institute of the Russian Academy of Science (Rozhdestvensky 1964, 1968) and then later (1961–1967) prospecting and excavations were made by teams from the Laboratory of Paleobiology of the Kazakh Academy of Sciences (Nurumov 1964; Bazhanov 1972; Nesov and Khisarova 1988). Nesov collected vertebrates at Shakh Shakh in 1980 and 1982. In 1995 and 2002–2007 the locality was sampled by international expeditions (Kordikova et al. 2001; Dyke and Malakhov 2004; Malakhov et al. 2009; Averianov et al. 2014). Additional specimens were also collected at this site in 2012 (Syromyatnikova and Danilov 2013).

The section of the Bostobe Formation at Shakh Shakh is composed of alternating predominantly red clays and sandstones. The main fossiliferous bed is the red clay in the middle of the section (Rozhdestvensky 1964).

The revised vertebrate fauna known from Shakh Shakh includes the euryhaline chondrychthians Polyacrodus cf. brabanticus, *Hybodus kansaiensis* and *Myledaphus glickmani*, chondrosteans (Acipenseridae indet.), holosteans (Amiidae indet.), teleosteans (Aspidorhynchiformes indet., Ichthyodectidae indet.), cryptobranchids (*Eoscapherpeton* sp.), the possible proteid *Bishara backa*, anurans (Discoglossidae indet.), the macrobaenid *Anatolemys maximus*, the adocids *Shachemys baibolatica*, *Adocus bostobensis*, trionychids (*Aspideretoides riabinini* and “Trionyx” kansaiensis), the stem testudinoid *Lindholmemys gravis*, squamates (Scincomorpha indet.), crocodyliforms cf. *Kansajsuchus* sp. and Eusuchia indet., Ankylosauridae indet., the lambeosaurine *Aralosaurus tuberiferus*, Sauropoda indet., Ornithomimidae indet., Tyrannosauroidea indet., Therizinosauroidea indet., Caenagnathidae (?) indet., birds and eutherian mammals (*Beleutinus orlovi* and *Zhalmouzia bazhanovi*) (Rozhdestvensky 1964; Rozhdestvensky and Khozatsky 1967; Rozhdestvensky 1968, 1970, 1973; Kuznetsov 1976, 1977; Glickman 1980; Suslov 1982; Mertinene and Nesov 1985; Nesov 1986; Kuznetsov and Chkhikvadze 1987; Efimov 1988; Nesov and Khisarova 1988; Nesov 1995; Storrs and Efimov 2000; Kordikova et al. 2001; Dyke and Malakhov 2004; Godefroit et al. 2004; Averianov 2007b; Danilov et al. 2007; Syromyatnikova and Danilov 2009; Vitek and Danilov 2010; Danilov et al. 2011; Averianov and Sues 2012; Skutschas 2013; Syromyatnikova and Danilov 2013; Averianov et al. 2014).

**Figure 3.**
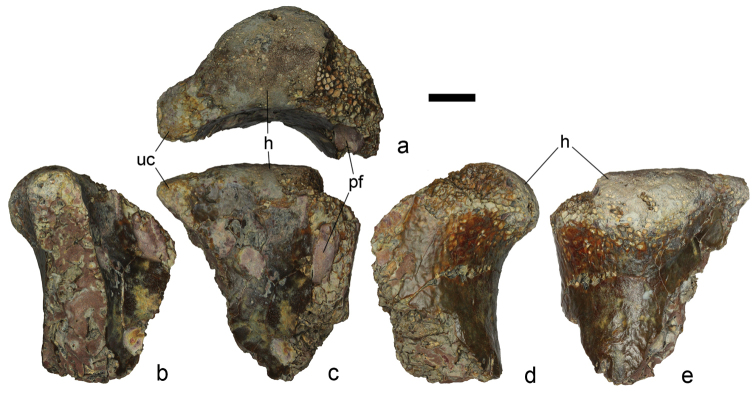
*Aralazhdarcho bostobensis*, ZIN PH 57/43, a proximal fragment of a left humerus in proximal (**a**), ventral (**b**), anterior (**c**), dorsal (**d**), and posterior (**e**) views. This specimen is from the Shakh Shakh II locality in the northeasten Aral Sea region of Kazakhstan; Bostobe Formation, Upper Cretaceous (Santonian – lower Campanian). Abbreviations: h, humeral head; pf, pneumatic foramen; uc, ulnar crest. Scale bar is 10 mm.

The first pterosaur bones reported from Shakh Shakh were collected by Rozhdestvensky but not recognized as such at the time (Averianov 2004: fig. 8). The first reported pterosaur bone from this locality is a jugal fragment (CCMGE 41/11915) collected by Nesov in 1982 (Nesov 1984: pl. 7, fig. 13, 1990, 1997: pl. 15, fig. 13; Averianov 2004: fig. 7c-e). All identifiable pterosaur specimens from Shakh Shakh likely belong to an azhdarchid which was subsequently described as *Aralazhdarcho bostobensis* (Averianov 2007a: pl. 9). Described referred specimens include an edentulous jaw fragment (ZIN PH 37/43), an anterior fragment of a mid-cervical vertebra (ZIN PH 9/43), an atlas–axis centrum (ZIN PH 44/43), a posterior dorsal vertebral centrum (ZIN PH 46/43), a distal fragment of a scapula (ZIN PH 45/43), the proximal end of a wing phalanx 2 (ZIN PH 16/43), and a proximal fragment of femur (ZIN PH 43/43). In 2007 and 2012 some additional but poorly preserved pterosaur bones were also collected by our field teams. Of these remains, the best preserved new specimen is a proximal humerus fragment (ZIN PH 57/43) that was collected in 2012 (Fig. 3). The humeral head is incompletely preserved; it is crescentic in proximal view, with broadly concave anterior and pointed convex posterior sides (Fig. 3a). The most striking feature of this specimen is that the humeral head is not saddle-shaped, as in other azhdarchids (Averianov 2010); it is convex in anteroposterior section and almost flat in dorsoventral section as opposed to the concave shape seen in other azhdarchids (and an elevated dorsal ridge might be present on the missing dorsal part of the head) (Fig. 3a). The dorsal side of the proximal humerus extends anteriorly beyond the level of the humeral head but the base of the deltopectoral crest is missing on this specimen. This basal region should be distal to a huge pneumatic foramen on the anterior side (length 10.0 mm, width 5.5 mm; Fig. 3a, c) but only the base of the ulnar crest is preserved and directed ventrally, as in other pterodactyloids. Bennett (1989) considered the ventral (=posterior in non-flight position) orientation of the ulnar crest to be a synapomorphy for Ornithocheiroidea (Pteranodontidae + Ornithocheiridae) but this is based on comparison with the holotype of *Bennettazhia oregonensis*, where the ulnar crest is actually missing. The anterior side of this bone is deeply concave between the pneumatic foramen and the base of ulnar crest in dorsoventral section and sinusoidal anteroposteriorly. The opposite posterior side is convex in dorsoventral section and concave anteroposteriorly. The humeral neck is inclined posteriorly to the long axis of the shaft at an angle of ~40° while the articular surface of the humeral head overhangs the neck along the posterior side, so it is exposed posteriorly much more than anteriorly (Fig. 3e).

## Paleoenvironments

**Figure 4.**
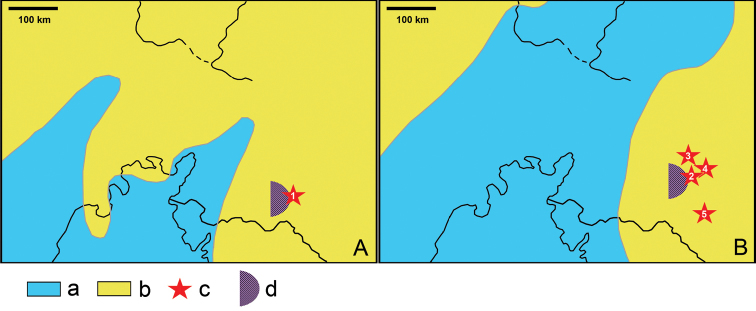
Relative positions of known pterosaur localities of the northeastern Aral Sea region of Kazakhstan in Turonian (**A**) and Santonian (**B**) times. These paleogeographic maps are modified from Martinson and Nikitin (1978). Legend: a, sea; b, land; c, pterosaur locality (**1** Tyulkili; **2** Baibishe; **3** Akkurgan; **4** Buroinak; **5** Shakh-Shakh); d, estuarian paleoenvironment indicated by marine sharks.

**Figure 5.**
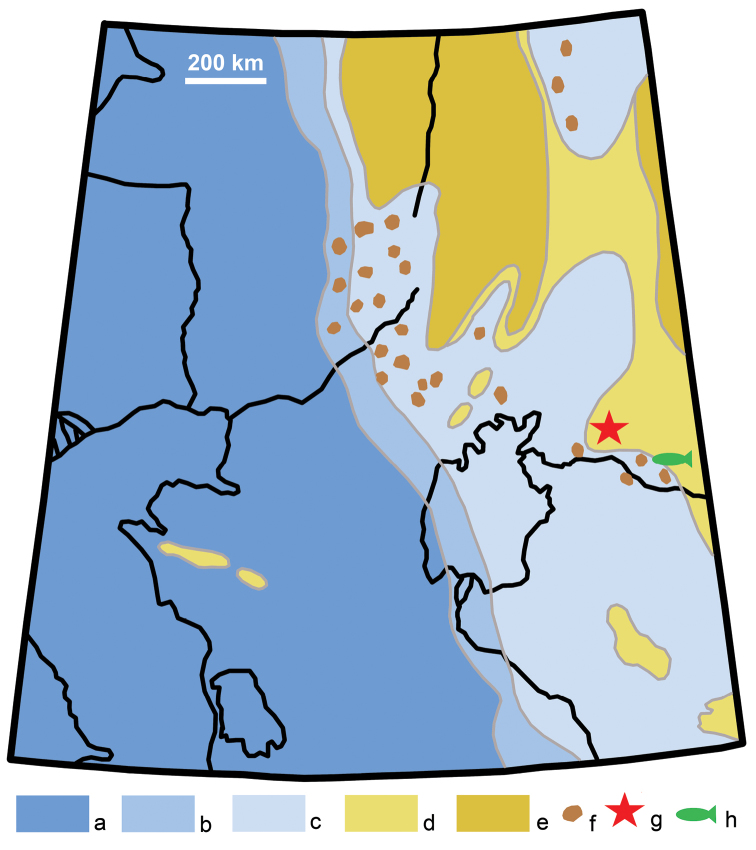
Paleogeographic map of western Kazakhstan in the Santonian-Campanian, modified and simplified from Sagunov and Tkachev (1975: fig. 23). Legend: a, deep water sea; b, shelf; c, shallow water sea; d, lowland coastal plain; e, elevated land; f, area of phosphate accumulation; g, pterosaur locality; h, clupeomorph locality (Taldysai).

The northeastern Aral Sea region, which is now in the centre of Asia, was located for most of the Late Cretaceous on the westernmost periphery of the ancient Asian landmass and was bordered by the Turgai Strait which connected Tethys to the Arctic ocean. The Turgai Sea, an infilling of the Turgai Depression north of the Aral Sea, extended north and retreated south several times following fluctuations in sea levels. In particular, the Turgai retreated during the Cenomanian regression but partially returned during the early Turonian transgression (Geology of the USSR 1970; Martinson and Nikitin 1978) (Fig. 4A). The next phase of transgression was in the Santonian to early Campanian when the Turgai Sea extended further north (Figs 4B, 5).

In paleoclimatic reconstructions, the northeastern Aral Sea region is placed at the border of tropical and subtropical climate zones in the Turonian and Santonian (Semikhatov and Chumakov 2004). Abundant remains of crocodyliforms in both the Zhirkindek and Bostobe formations are also good indicators of tropical-to-subtropical climatic conditions (Markwick 1998). Under subtropical or tropical climatic conditions in the Santonian, the western coast of the Asian landmass in this region was likely affected by strong winds, which caused significant aridization of the climate as evidenced by paleobotanical data (see below) on the one hand and steady upwelling of deep sea waters along the coast on the other. We know that the Santonian–early Campanian was a time of intensive phosporite accumulation in the shallow waters of this region (Fig. 5), including the Aktobe phosphorite basin to the north of the Aral Sea, which occupies 25,000–30,000 km^2^ and has resources of 700 million tons of P_2_O_5_ (Zhelezko 1987). Because of this high concentration of phosphorus and other minerals elevated from the sea bottom, upwelling areas are “oases of life,” places of high bioproductivity in terms of microorganisms and their consumers: primary (i.e., crustaceans and other marine invertebrates), secondary (fishes), and tertiary (i.e., marine reptiles, mammals, and sea birds). In the modern oceans, upwelling areas occupy only about 5% of total global area but produce about 25% of all fisheries (Jennings et al. 2001). There is no doubt that upwelling areas were also important in the Mesozoic. The Aktobe area produces numerous phosphatized shark teeth and fish bones (Zhelezko 1987), as well as remains of marine reptiles. Such abundant fish resources likely attracted pterosaurs that may have spent some of their time around the coast line (Fig. 5).

Localities within the Zhirkindek and Bostobe formations also contain the abundant remains of a few species of chondrychthians, amongst which the most common are hybodontiform sharks (*Hybodus kansaiensis* and *Polyacrodus* sp.) and the rhinobatoid skate *Myledaphus glickmani* (Nesov and Mertinene 1986; Nesov 1988a, 1997; Nesov and Khisarova 1988). Nesov considered this to be evidence that these accumulation basins were estuaries. Hybodontiform sharks and *Myledaphus* are common in Late Cretaceous fluvial deposits (Estes 1964; Peng et al. 2001; Neuman and Brinkman 2005) and it cannot be excluded that these taxa may have also entered freshwater rivers. Indeed, the presence of the marine lamnoid sharks *Protolamna* sp. and *Cretodus longiplicatus* within certain parts of the Zhirkindek Formation at the Tyulkili section clearly indicates ingression of sea waters in the basin. In the Bostobe Formation the Baibishe locality has the highest diversity of chondrychthians and includes taxa not found in other Bostobe localities (*Scapanorhynchus* sp., *Ischyrhiza* sp., *Protoplatyrhina* sp., *Ptychotrigon* sp.). Among these taxa, the sclerorhynchid skate *Ischyrhiza* may also occur in fluvial deposits (Estes 1964; Peng et al. 2001) and the mitsukurinid lamnoid shark *Scapanorhynchus* also occurs in the fluvial Turonian Bissekty Formation of the Kyzylkum Desert, Uzbekistan (unpublished data). Modern goblin sharks (*Mitsukurina*) are deep water (mesopelagic) species (Yano et al. 2007) yet their closest extinct relative *Scapanorhynchus* is common in near shore Late Cretaceous deposits (Arambourg 1952; Bardet et al. 2000; Kennedy et al. 2008). Our view is that the Baibishe locality likely formed in an estuarine basin while the other known Bostobe Formation vertebrate localities are of fluvial origin (Dyke and Malakhov 2004).

The mollusc fauna of the Zhirkindek and Bostobe formations consists of few unidentified gastropods along with numerous and diverse bivalves. The majority of the bivalves (19 species) belong to two orders, Unionida and Trigoniida, with a single species belonging to the order Veneroidei. Unionida are a numerous and widely distributed group well-represented in modern faunas, while the Trigoniida were more diversified and successful in the past. Just the single trigoniid genus *Neotrigonia* occurs today along with a few species of marine clams that inhabit the Australian coast from below tide level to at least 400 m water depth (Stanley 1984). The single veneroidean species, *Limnocyrena tasaranica*, is from the Cyrenoididae, which is represented in the modern fauna by brackish water species, like the Florida marsh clam, *Cyrenoida floridana*, which lives in Florida Bay estuaries under salinity of 0–18‰ (Brewster-Wingari and Ishman 1999). The *Pseudohyria*-*Sainshandia*-*Limnocyrena* association found in the Zhirkindek Formation is widely distributed in north China in formations of Campanian-Maastrichtian age (Ma 1994).

In the paleontological literature Cretaceous trigoniid molluscs, generally referred to as Trigonioidoidea, are usually described as being freshwater (Martinson 1957; Tumpeesuwan et al. 2010) or non-marine in their mode-of-life (Martinson 1990a, b, 1997; Ma 1994; Chen et al. 2006; Komatsu et al. 2007; Sha 2007, 2010). This is interesting because the only living genus of the group, *Neotrigonia*, is marine (see above). Martinson (1957) thought that large, thick-shelled and sculptured *Trigonioides* and *Sainshandia* lived in habitats transitional between freshwater and marine, most likely in lagoons. Based on its shell microstructure, Kolesnikov (1977) concluded that *Pseudohyria* (known from lower and upper Cretaceous deposits in Mongolia) lived in shallow areas of large lakes that had increased salinity and alkalinity in a warm and arid climate. However, according to Martinson (1982), fossil trigoniid molluscs were psammophilous, living in sandy ground in the highly energetic tidal zone of large lakes. Indeed, the thick and sculptured shells of trigoniid bivalves are reminiscent of marine bivalves which live in the marine tidal zone. Their massive sculptured shells may also be due to the large amounts of free calcium available in arid tropical and subtropical basins. These bivalves dominated in such energetic environments while gastropods are very rare; this latter group tend to be much more common in shallow bays that have lower energy waters (Martinson 1982, 1990a). This possible correlation might explain the rarity of fossil gastropod remains in the Zhirkindek and Bostobe formations.

The flora of the Turonian Zhirkindek Formation at the Kankazgan [=Tyulkili] locality is dominated by conifers alongside less abundant angiosperms and ferns (Shilin 1998). In contrast, the known flora at KIarakumzholy (within the same Formation) is dominated by angiosperms and a few ferns while conifers are so far absent (Shilin 2008). This association of conifers, ferns and angiosperms is characteristic of estuarine marshy environments in western Europe (Coiffard et al. 2012) but other gymnosperms are also common. An estuarine marshy paleoenvironmental interpretation for the Kankazgan locality is likely and is supported by the discovery of the marine shark *Cretodus longiplicatus*. This locality was close to the coastline in the Turonian (Fig. 4b), while KIarakumzholy, located at the eastern end of Tyulkili hills, was more inland, and its floral composition with angiosperms and ferns corresponds to Cenomanian-Santonian floodplain environments in western Europe (Coiffard et al. 2012).

According to Shilin and Romanova (1978: tab. 1) at two plant localities within the Santonian – early Campanian Bostobe Formation, Shakh Shakh and Taldysai, the flora consists only of conifers (24.2% from 227 specimens) and angiosperms (75.8%). The absence of ferns, cycads and ginkgo is striking (Shilin 1971) although both these groups were later reported in the Shakh Shakh-Taldysai floral stage (Shilin 1998). In the Cretaceous of western Europe the association of abundant conifers and angiosperms is only characteristic of Cenomanian brackish marsh environments (Coiffard et al. 2012), alongside other abundant gymnosperms. We note that Taldysai is located in the Zhezkazgan-Sarysu Depression (Fig. 5h) and has also yielded the abundant remains of trigonioidid bivalves, insects, possible euryhaline herrings referred to *Diplomystus* (Khisarova 1972, 1974) (best classified as Clupeomorpha indet.; Grande 1985), and bird feathers (Shilin and Romanova 1978: pl. 26, figs 5-7; Nesov 1992a). All this is consistent with a brackish marsh paleoenvironmental interpretation.

The known flora of the Bostobe Formation (Shakh Shakh and Taldysai) includes 8 species of ferns and 40 species of angiosperms (Shilin 1971; Shilin and Romanova 1978). Of these, the angiosperms are represented by narrow-leaved and small-leaved deciduous and subtropical trees and shrubs. The Santonian-Campanian flora of this region differs from the Turonian flora discussed above because of the absence (or rarity) of cycads and absence of large-leaved angiosperm which might indicate more arid conditions (Shilin and Romanova 1978). Increased aridization in this region is corroborated by pollen samples from these Santonian-Campanian deposits which contain abundant xerophylous taxa (Shilin and Romanova 1978).

## Conclusions

All so far identifiable pterosaur remains from the Zhirkindek and Bostobe formations of Kazakhstan appear, based on present evidence, to belong to Azhdarchidae (although many specimens, as we have reviewed, are very incompletely preserved): *Azhdarcho* sp. is known from the Zhirkindek Formation while *Aralazhdarcho bostobensis* (and *Samrukia nessovi*) are known from the Bostobe Formation. As noted, and despite a large size difference, the possibility remains that *Samrukia nessovi* is a synonym of *Aralazhdarcho bostobensis*. Based on other palaeontological and palynological evidence, we suggest that the azhdarchid pterosaurs from the Tyulkili locality (Zhirkindek Formation) lived in a tropical-to-subtropical relatively humid climate on the shore of an estuarine basin connected to the Turgai Sea. Evidence from bivalves and the presence of marine sharks (*Cretodus* and *Protolamna*) supports the argument that this basin was subject to periodic influxes of sea water and at times reached normal marine salinity. The flora is dominated by thermophilous conifers and less abundant ferns and angiosperms which indicate an estuary marsh paleoenvironment. The azhdarchids from the Bostobe Formation lived on the coastal fluvial plain along the Turgai Sea. Most localities (Akkurgan, Buroinak, and Shakh Shakh) were formed in a floodplain environment but the sediments at the Baibishe locality were formed in a brackish-water estuary as indicated by a greater diversity of chondrychthians and brackish-water bivalves. The shallow waters near this coastal flood plain were a place of intensive phosphate accumulation due to upwelling conditions caused by strong winds from the ancient Asian landmass. These winds were also the reason for significant aridization during the Santonian and early Campanian, which is evident from paleobotanical data. We speculate that pterosaurs may have been attracted to this area by an abundance of resources in the highly bioproductive estuaries and nearshore upwelling waters.
